# Inflammation mediates the association between hyperuricemia and stroke mortality: a cohort study

**DOI:** 10.3389/fneur.2025.1599730

**Published:** 2025-08-01

**Authors:** Yiwen He, Jun You, Zhenjie Fan, Zhiyong Wang, Min Qian

**Affiliations:** ^1^The First College of Clinical Medical Science, China Three Gorges University, Yichang, China; ^2^Department of Emergency, Yichang Central People’s Hospital, Yichang, China

**Keywords:** hyperuricemia, stroke, mortality, inflammation, NHANES, mediation analysis

## Abstract

**Background:**

Stroke is a leading cause of mortality and disability worldwide. The relationship between hyperuricemia and stroke prognosis remains controversial. This study aims to investigate the association between hyperuricemia and stroke prevalence, as well as the impact of hyperuricemia on mortality risk among stroke patients, utilizing data from the National Health and Nutrition Examination Survey (NHANES) 2001–2018.

**Methods:**

Hyperuricemia was defined as serum uric acid ≥416 μmol/L (7.0 mg/dL) in men or ≥357 μmol/L (6.0 mg/dL) in women. We conducted weighted logistic regression analyses to assess the association between hyperuricemia and stroke prevalence. Cox proportional hazards regression models were used to evaluate the impact of hyperuricemia on all-cause and cardiovascular mortality among stroke patients. The models were progressively adjusted for demographic factors, lifestyle factors, comorbidities, and biomarkers. Time-dependent ROC curves were constructed to assess predictive performance. Restricted cubic splines were applied to investigate potential nonlinear relationships between serum uric acid and mortality. Subgroup and mediation analyses explored the interactions and indirect effects, respectively. Sensitivity analyses were conducted to ensure the robustness of the results.

**Results:**

Hyperuricemia was associated with increased odds of stroke (adjusted OR 1.25; 95% CI: 1.07–1.45; *p* = 0.005). Among 1,579 stroke patients, hyperuricemia was linked to higher risks of all-cause mortality (adjusted HR 1.25; 95% CI: 1.06–1.48; *p* = 0.008) and cardio-cerebrovascular mortality (adjusted HR 1.38; 95% CI: 1.05–1.80; *p* = 0.020). Inflammation markers SII and CRP partially mediated these associations.

**Conclusion:**

Hyperuricemia is associated with an increased prevalence of stroke. It is also strongly associated with increased mortality in stroke patients, an association mediated in part by inflammation.

## Background

Globally, stroke ranks as the second leading cause of death and a major contributor to mortality and disability (1). In China, stroke has become the foremost cause of death and disability (2). Only a small proportion of patients receive effective treatment within the eligible therapeutic time window (such as intravenous thrombolysis or endovascular thrombectomy) (3). Recent studies indicate that since 2015, the decline in stroke incidence has plateaued, and the prevalence and mortality rates have even increased in regions like Southeast Asia, East Asia, Oceania, countries with lower socio-demographic indices, and among individuals under 70 years old (4). Stroke imposes a significant disease burden on global health, individual families, and societal medical costs (5, 6).

In addition to traditional risk factors, the role of emerging metabolic risk factors in the development and progression of stroke is increasingly recognized. Among these, hyperuricemia—a chronic metabolic disorder resulting from purine metabolism dysfunction—is showing a rising prevalence trend (7). It has been associated with various diseases, including diabetes (8), gout (9), hypertension (10), coronary heart disease (CHD) (11), metabolic disorders, and kidney diseases (12). Moreover, the link between hyperuricemia and mortality risk has garnered growing attention (13–15). However, the relationship between serum uric acid (SUA) levels and cardiovascular diseases, including stroke, remains a subject of significant controversy. Some studies suggest no association, while others even indicate an inverse relationship (16, 17). Particularly in the field of stroke, the impact of SUA on the prognosis of stroke patients (e.g., mortality risk) remains a subject of intense debate (18), with some studies suggesting no association, while others support a neuroprotective role (19, 20).

Therefore, this study aims to utilize the large-scale cohort data from the National Health and Nutrition Examination Survey (NHANES) to investigate the following critical questions, addressing current knowledge gaps: (1) The association between hyperuricemia and stroke prevalence in the general population; (2) The impact of hyperuricemia on all-cause mortality and cardiovascular-specific mortality among patients diagnosed with stroke. By gaining deeper insights into the role of hyperuricemia in the long-term prognosis of stroke patients—particularly in resolving the aforementioned key controversies—we aim to provide a more robust scientific basis for clinical risk stratification and targeted interventions.

## Methods

### Study population

A large-scale study designed to assess the health and nutritional status of the U.S. population, NHANES includes data on demographics, physical examinations, questionnaires, and laboratory tests. NHANES data can be linked to mortality data from the National Center for Health Statistics (NCHS) up to December 31, 2019, allowing us to conduct cohort studies.

**Figure 1:**
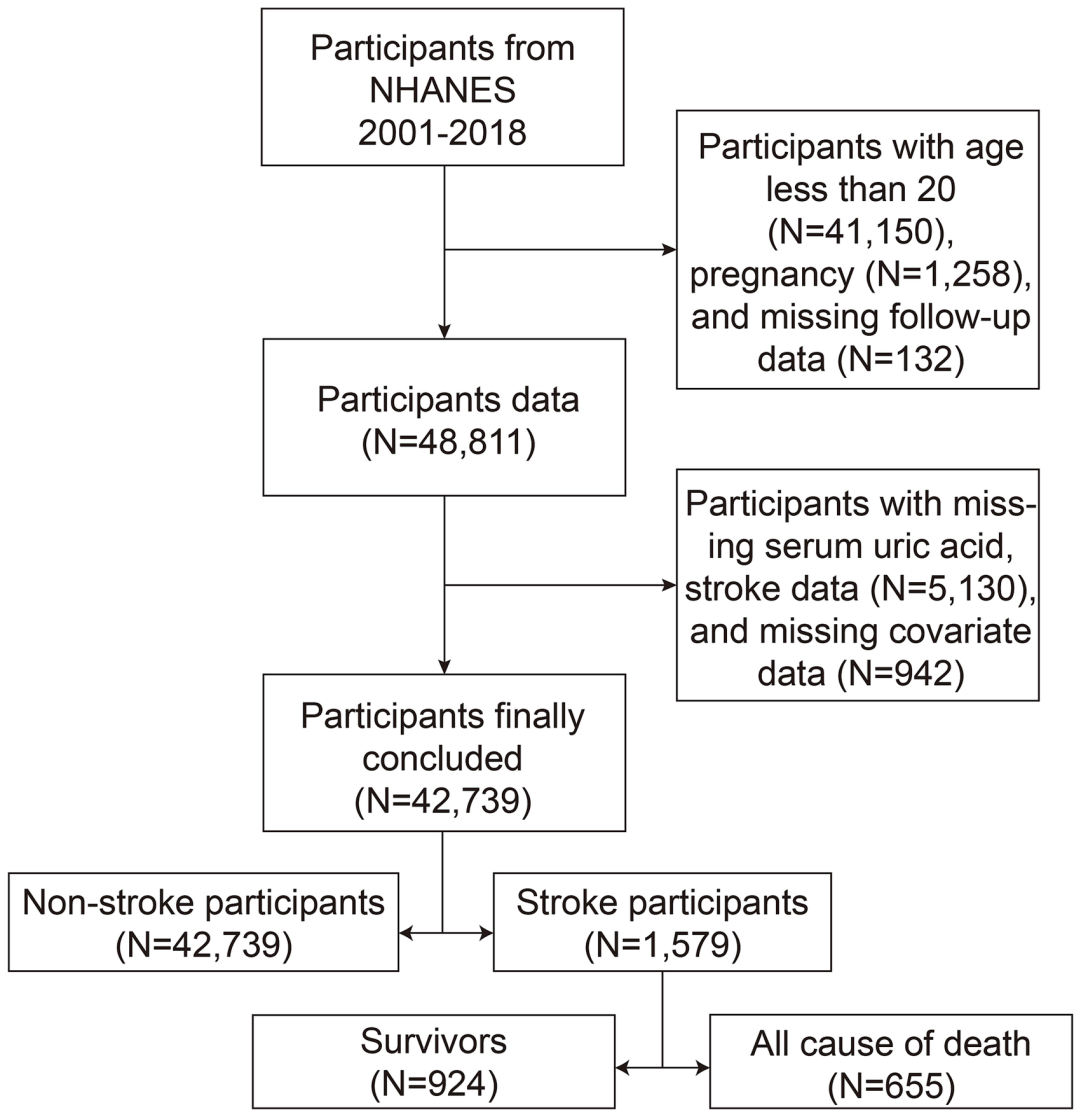
Inclusion and exclusion flowchart. NHANES, National Health and Nutrition Examination Survey.

This study utilized NHANES data from 2001 to 2018, focusing on adults; thus, minors were excluded. Additionally, participants lacking serum uric acid data, stroke data, or who were pregnant or lost to follow-up were excluded (Figure 1). Missing data were handled by case-wise exclusion: any participant with missing values in key variables (including serum uric acid, stroke status, or covariates required for regression models) was removed from the final analysis. All participants gave their informed consent, and the study received approval from the NCHS Institutional Review Board. Consequently, no further consent or ethical approval was necessary. All procedures adhered to the applicable guidelines and regulations.

### Definition of hyperuricemia and stroke

Serum uric acid (SUA) concentration was determined using the standardized NHANES laboratory protocol (DxC800 analyzer, timed endpoint method; detailed procedure: https://wwwn.cdc.gov/Nchs/Data/Nhanes/Public/2011/DataFiles/BIOPRO_G.htm#LBXSUA). To ensure data quality, observations with SUA values below the lower limit of detection (0.4 mg/dL) or above the upper limit of detection (11.3 mg/dL) were excluded from analysis.

Participants were considered to have hyperuricemia if their SUA levels were ≥416 μmol/L (7.0 mg/dL) for men and ≥357 μmol/L (6.0 mg/dL) for women. Stroke was defined based on the questionnaire question: “Has a doctor or other health professional ever told you that you have had a stroke?” (21, 22).

### Covariates

Potential confounding variables were selected, including demographic factors (age, sex, race, poverty level, education level, and marital status), lifestyle factors (smoking and drinking), baseline disease conditions (CHD, diabetes, cancer, and hyperlipidemia), and biomarkers [total cholesterol (TC), aspartate aminotransferase (AST), alanine aminotransferase (ALT), albumin, monocytes, neutrophils, lymphocytes, and platelet count].

### Statistical analysis

For continuous variables that followed a normal distribution, the data are shown as mean ± standard deviation (SD). For variables that were not normally distributed, the data are presented as the median with interquartile range. Categorical data are expressed as frequencies and percentages. Comparisons between groups were carried out using t-tests for normally distributed continuous variables, the Mann–Whitney U test for non-normally distributed variables, and Chi-square tests for categorical variables.

We conducted weighted logistic regression analyses with three stepwise-adjusted models to examine the association between hyperuricemia and stroke prevalence. In survival analysis, Kaplan–Meier curves were used to estimate survival probabilities, and the log-rank test was employed to compare survival differences between groups. Similar to the logistic regression, three stepwise-adjusted Cox proportional hazards regression models were established to evaluate the association between hyperuricemia and all-cause mortality and cardio-cerebrovascular disease mortality among stroke patients. Restricted cubic spline (RCS) analyses were employed to evaluate the nonlinear correlation between serum uric acid levels and mortality risk in patients who had experienced a stroke. Stratified by gender, with the likelihood ratio test used to assess nonlinearity. To evaluate the predictive performance of hyperuricemia on mortality outcomes, time-dependent receiver operating characteristic (ROC) curve analyses were performed, calculating the area under the curve (AUC) at specific clinically relevant time points: 3 years, 5 years, and 10 years after the baseline assessment. These time points were selected based on their established clinical significance in long-term mortality follow-up studies for stroke patients and to assess both intermediate (3–5 years) and long-term (10 years) predictive ability.

Subgroup analyses were conducted to assess potential interactions between hyperuricemia and various subgroups. Mediation analysis was performed to calculate the average causal mediation effect (ACME), average direct effect (ADE), and proportion mediated, exploring possible factors mediating the relationship between hyperuricemia and mortality risk in stroke patients. Finally, sensitivity analyses were conducted to assess the robustness of the results.

All statistical analyses were performed using R software (version 4.3.3). All statistical tests were two-sided, and a *p*-value of less than 0.05 was considered statistically significant.

## Results

### Baseline characteristics

This study included 32,766 participants, among whom 1,579 were identified as having had a stroke (Table 1). Stroke participants were significantly older than those in the non-stroke group. The proportion of females and non-Hispanic Black participants was higher in the stroke group, while Mexican Americans had a higher proportion in the non-stroke group. Additionally, the stroke group had higher prevalence rates of hyperuricemia (31%), hypertension (78.3%), CHD (18.9%), diabetes (35.8%), and cancer (22.2%), all with *p*-values less than 0.001. Biochemical indicators showed that the stroke group had higher systemic immune-inflammation index (SII) and SUA levels.

**Table 1:** Weighted basic characteristics of participants according to presence or absence of stroke.

Characteristics	Non-stroke (*n* = 42,739)	Stroke (*n* = 1,579)	*p* value
Sex (%)			<0.001
Female	50.9	57.8	
Male	49.1	42.2	
Age (%)			<0.001
Age < 50	57.4	16	
Age 50–65	25.8	27.9	
Age > 65	16.8	56	
Race (%)			<0.001
Mexican American	8.4	4.3	
Non-Hispanic White	68.6	71.5	
Non-Hispanic Black	10.5	14.3	
Other	12.5	9.9	
Poverty level (%)			<0.001
Non-poverty	87.4	81.7	
Poverty	12.6	18.3	
Marital status (%)			<0.001
No	35.7	43.3	
Yes	64.3	56.7	
Education level (%)			<0.001
High School or above	16.1	28.2	
Below high school	83.9	71.8	
Smoking (%)			0.002
No	78.8	74.4	
Yes	21.2	25.6	
Drinking (%)			<0.001
No	36.7	43	
Yes	63.3	57	
Hyperuricemia (%)			<0.001
No	81.6	69	
Yes	18.4	31	
Hypertension (%)			<0.001
No	64	21.7	
Yes	36	78.3	
CHD (%)			<0.001
No	96.9	81.1	
Yes	3.1	18.9	
Diabetes (%)			<0.001
No	87.5	64.2	
Yes	12.5	35.8	
Cancer (%)			<0.001
No	90.8	77.8	
Yes	9.2	22.2	
BMI, kg/m^2^	27.70 [24.08;32.20]	28.92 [25.10;33.70]	<0.001
SUA, μmol/L	315.20 [261.70;374.70]	333.10 [273.60;404.50]	<0.001
TC, mg/dL	193.00 [167.00;220.00]	184.00 [157.00;216.00]	<0.001
AST, U/L	23.00 [19.00;27.00]	22.00 [19.00;27.00]	0.032
ALT, U/L	21.00 [16.00;29.00]	19.00 [15.00;26.00]	<0.001
Albumin, g/dL	43.00 [41.00;45.00]	41.00 [39.00;44.00]	<0.001
Monocyte, 1,000 cells/μL	0.50 [0.40;0.70]	0.60 [0.50;0.70]	<0.001
Neutrophils, 1,000 cells/μL	4.00 [3.10;5.10]	4.33 [3.40;5.50]	<0.001
Lymphocyte, 1,000 cells/μL	2.00 [1.60;2.50]	1.90 [1.50;2.40]	<0.001
Platelet, 1,000 cells/μL	245.00 [209.00;290.00]	233.62 [194.00;280.00]	<0.001
SII	482.62 [350.24;673.06]	523.22 [370.23;728.36]	<0.001

The continuous variables were presented as either the mean ± standard deviation or the median with interquartile range, and *p* values were calculated using weighted t-tests for normally distributed variables and weighted Mann–Whitney U tests for non-normally distributed variables. Categorical variables were expressed as weighted percentages and *p* values were computed using weighted chi-square tests. BMI, body mass index; SUA, serum uric acid; TC, total cholesterol; AST, aspartate aminotransferase; ALT, alanine aminotransferase; SII, systemic immune-inflammation index; CHD, coronary heart disease.

Table 2 shows the baseline characteristics of 1,579 stroke patients, classified according to their survival status. By the end of follow-up on December 31, 2019, there were 635 all-cause mortality events, with a median survival time of 74.0 [35.5; 119] months. Among stroke patients, those who died were often older (82% were over 65 years old), more likely to be male, and non-Hispanic Black participants. Deceased patients had higher prevalence rates of hyperuricemia, hypertension, CHD, and cancer. Notably, deceased stroke patients had significantly higher SII and SUA levels (*p* < 0.001).

**Table 2:** Basic characteristics of stroke participants grouped according to survival status.

Characteristics	Total (*N* = 1,579)	All-cause mortality		*p* value
		No (*N* = 924)	Yes (*N* = 655)	
Sex (%)				0.001
Female	813 (51.5%)	523 (56.6%)	290 (44.3%)	
Male	766 (48.5%)	401 (43.4%)	365 (55.7%)	
Age (%)				<0.001
Age < 50	186 (11.8%)	166 (18.0%)	20 (3.05%)	
Age 50–65	430 (27.2%)	332 (35.9%)	98 (15.0%)	
Age > 65	963 (61.0%)	426 (46.1%)	537 (82.0%)	
Race (%)				<0.001
Mexican American	158 (10.0%)	102 (11.0%)	56 (8.55%)	
Non-Hispanic White	413 (26.2%)	280 (30.3%)	133 (20.3%)	
Non-Hispanic Black	826 (52.3%)	401 (43.4%)	425 (64.9%)	
Other	182 (11.5%)	141 (15.3%)	41 (6.26%)	
Poverty level (%)				0.009
Non-poverty	1,220 (77.3%)	692 (74.9%)	528 (80.6%)	
Poverty	359 (22.7%)	232 (25.1%)	127 (19.4%)	
Marital status (%)				0.032
No	763 (48.3%)	425 (46.0%)	338 (51.6%)	
Yes	816 (51.7%)	499 (54.0%)	317 (48.4%)	
Education level (%)				0.001
High School or above	569 (36.0%)	294 (31.8%)	275 (42.0%)	
Below high school	1,010 (64.0%)	630 (68.2%)	380 (58.0%)	
Smoking (%)				0.001
No	1,203 (76.2%)	673 (72.8%)	530 (80.9%)	
Yes	376 (23.8%)	251 (27.2%)	125 (19.1%)	
Drinking (%)				<0.001
No	702 (44.5%)	372 (40.3%)	330 (50.4%)	
Yes	877 (55.5%)	552 (59.7%)	325 (49.6%)	
Hyperuricemia (%)				<0.001
No	1,071 (67.8%)	660 (71.4%)	411 (62.7%)	
Yes	508 (32.2%)	264 (28.6%)	244 (37.3%)	
Hypertension (%)				0.002
No	303 (19.2%)	202 (21.9%)	101 (15.4%)	
Yes	1,276 (80.8%)	722 (78.1%)	554 (84.6%)	
CHD (%)				<0.001
No	1,293 (81.9%)	790 (85.5%)	503 (76.8%)	
Yes	286 (18.1%)	134 (14.5%)	152 (23.2%)	
Diabetes (%)				0.130
No	957 (60.6%)	575 (62.2%)	382 (58.3%)	
Yes	622 (39.4%)	349 (37.8%)	273 (41.7%)	
Cancer (%)				<0.001
No	1,241 (78.6%)	768 (83.1%)	473 (72.2%)	
Yes	338 (21.4%)	156 (16.9%)	182 (27.8%)	
BMI, kg/m^2^	28.8 [25.1;33.2]	29.6 [25.6;34.3]	27.8 [24.6;31.8]	<0.001
SUA, μmol/L	339 [280;410]	327 [274;393]	357 [292;437]	<0.001
TC, mg/dL	183 [156;215]	185 [158;213]	180 [151;217]	0.312
AST, U/L	22.0 [19.0;27.0]	22.0 [18.0;27.0]	23.0 [19.0;28.0]	0.004
ALT, U/L	19.0 [15.0;25.0]	20.0 [15.0;26.0]	18.0 [15.0;24.0]	0.031
Albumin, g/dL	41.0 [39.0;43.0]	41.0 [39.0;43.0]	41.0 [38.0;43.0]	0.009
Monocyte, 1,000 cells/μL	0.60 [0.50;0.70]	0.60 [0.40;0.70]	0.60 [0.50;0.70]	<0.001
Neutrophils, 1,000 cells/μL	4.30 [3.30;5.40]	4.20 [3.20;5.40]	4.40 [3.40;5.50]	0.009
Lymphocyte, 1,000 cells/μL	1.90 [1.50;2.50]	2.00 [1.60;2.60]	1.80 [1.30;2.30]	<0.001
Platelet, 1,000 cells/μL	231 [193;276]	234 [198;278]	225 [185;272]	0.003
SII	512 [356;728]	488 [342;691]	553 [383;802]	<0.001

The continuous variables were presented as either the mean ± standard deviation or the median with interquartile range, and *p* values were calculated using t-tests for normally distributed variables and Mann–Whitney U tests for non-normally distributed variables. Categorical variables were expressed as percentages and frequencies, and *p* values were computed using weighted chi-square tests. BMI, body mass index; SUA, serum uric acid; TC, total cholesterol; AST, aspartate aminotransferase; ALT, alanine aminotransferase; SII, systemic immune-inflammation index; CHD, coronary heart disease.

### Association between hyperuricemia and stroke

In our three stepwise-adjusted weighted logistic regression models (Table 3), hyperuricemia consistently showed a positive association with the occurrence of stroke (all *p*-values < 0.05), indicating that patients with hyperuricemia were more likely to have a stroke. Specifically, the unadjusted Model 1 showed that hyperuricemia patients had approximately twice the odds of having a stroke compared to non-hyperuricemia patients (OR 1.98, 95% CI 1.73–2.27). As covariates were adjusted, the OR gradually decreased; in the fully adjusted Model 3, the OR decreased to 1.25 (OR 1.25, 95% CI 1.07–1.45), but the association remained significant.

**Table 3:** Weighted logistic regression analysis of hyperuricemia and stroke across three models.

Exposure	Model 1		Model 2		Model 3	
	OR (95%CI)	*p* value	OR (95%CI)	*p* value	OR (95%CI)	*p* value
Non-hyperuricemia	Reference		Reference		Reference	
Hyperuricemia	1.98 (1.73, 2.27)	<0.001	1.51 (1.31, 1.75)	<0.001	1.25 (1.07, 1.45)	0.005

Model 1, Did not adjust any covariates; Model 2, Adjusted for age, sex, and race; Model 3, Adjusted for sex, age, race, poverty level, marital status, education level, smoking, drinking, hypertension, coronary heart disease, diabetes, cancer, body mass index, total cholesterol, aspartate aminotransferase, alanine aminotransferase, albumin, monocytes, neutrophils, lymphocytes, and platelets; OR, odds ratio; CI, confidence interval.

### Hyperuricemia and survival status of stroke patients

**Figure 2:**
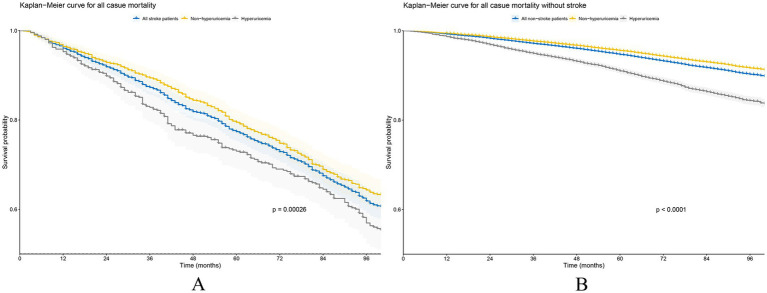
Kaplan–Meier survival curve analysis of all-cause mortality based on the presence or absence of hyperuricemia. **(A)** Stroke patients and **(B)** non-stroke patients.

The Kaplan–Meier survival curves (Figure 2), stratified by the presence of hyperuricemia, showed significant differences in survival probabilities between the stroke and non-stroke groups (log-rank test *p* < 0.05). Among stroke patients, the hyperuricemia group had significantly lower survival probabilities than the non-hyperuricemia group, suggesting that hyperuricemia is associated with poor prognosis in stroke patients. Further stepwise-adjusted Cox proportional hazards regression analyses (Table 4), hyperuricemia was found to be a significant predictor of mortality in stroke patients. In the unadjusted Model 1, there was a statistically significant association between hyperuricemia and an increased mortality risk, with an HR of 1.34 (95% CI 1.15–1.57). In Model 2, after adjusting for basic demographic factors, the risk remained significant but was slightly attenuated (HR 1.19, 95% CI 1.01–1.39). In the fully adjusted Model 3, after adjusting for more covariates, the HR slightly increased (HR 1.25, 95% CI 1.06–1.48). This may reflect the complex modulation of covariates on the relationship between hyperuricemia and mortality risk.

**Table 4:** Association between hyperuricemia and all-cause mortality and cardio-cerebrovascular diseases mortality in stroke patients.

Exposure	Model 1		Model 2		Model 3	
	HR (95%CI)	*p* value	HR (95%CI)	*p* value	HR (95%CI)	*p* value
All-cause mortality						
Non-hyperuricemia	Reference		Reference		Reference	
Hyperuricemia	1.34 (1.15, 1.57)	<0.001	1.19 (1.01, 1.39)	0.036	1.25 (1.06, 1.48)	0.008
Cardio-cerebrovascular diseases mortality						
Non-hyperuricemia	Reference		Reference		Reference	
Hyperuricemia	1.45 (1.12, 1.87)	0.005	1.30 (1.00, 1.68)	0.049	1.38 (1.05, 1.80)	0.020

Model 1, Did not adjust any covariates; Model 2, Adjusted for age, sex, and race; Model 3, Adjusted for sex, age, race, poverty level, marital status, education level, smoking, drinking, hypertension, coronary heart disease, diabetes, cancer, body mass index, total cholesterol, aspartate aminotransferase, alanine aminotransferase, albumin, monocytes, neutrophils, lymphocytes, and platelets; HR, hazard rate; CI; confidence interval.

**Figure 3:**
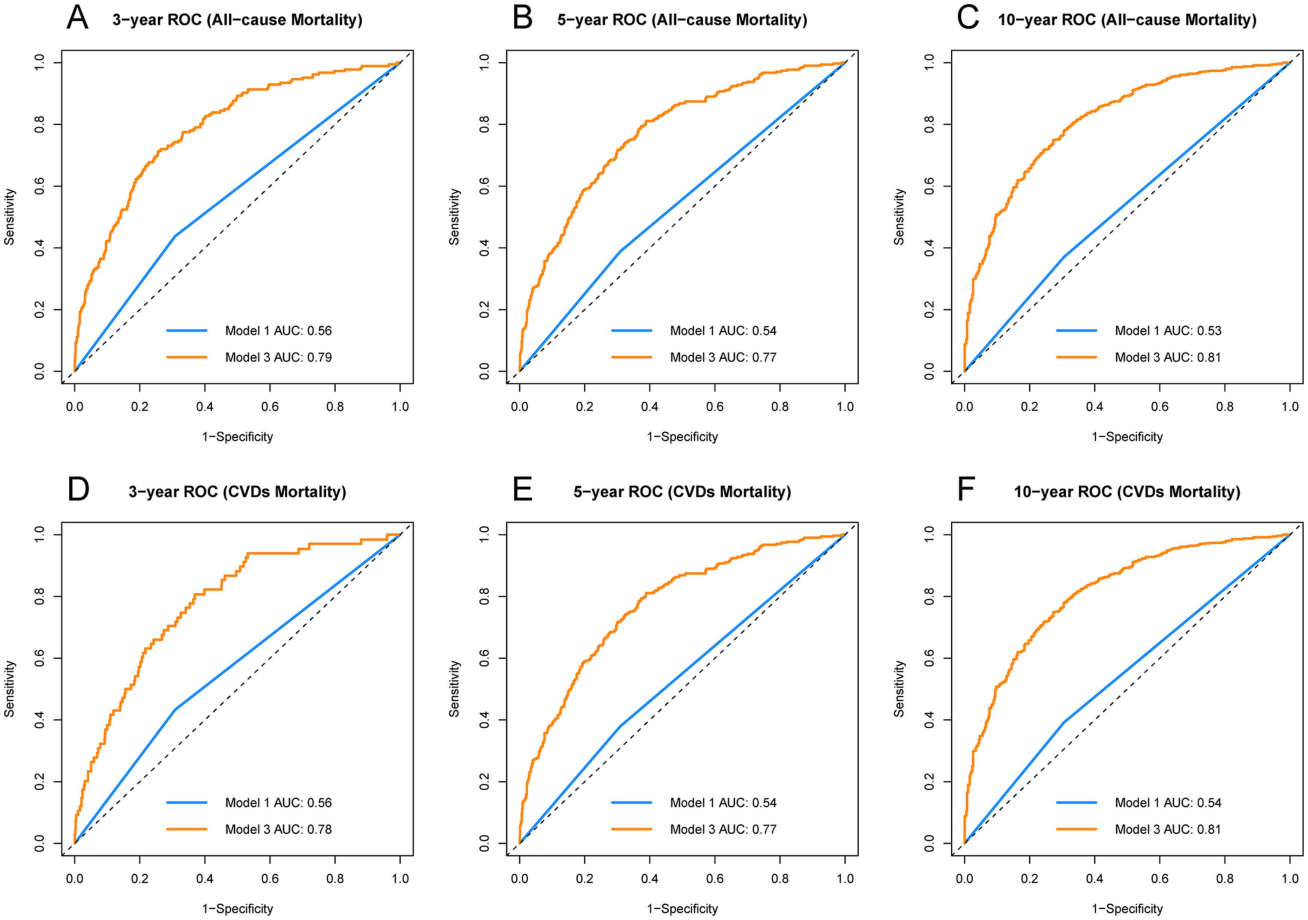
Comparative ROC curves for all-cause and cardio-cerebrovascular diseases mortality over different periods. **(A)** 3-year ROC curve for all-cause mortality, **(B)** 5-year ROC curve for all-cause mortality, **(C)** 10-year ROC curve for all-cause mortality, **(D)** 3-year ROC curve for CVDs mortality, **(E)** 5-year ROC curve for CVDs mortality, **(F)** 10-year ROC curve for CVDs mortality. ROC, receiver operating characteristic; AUC, area under the curve; CVDs, cardio-cerebrovascular diseases.

**Figure 4:**
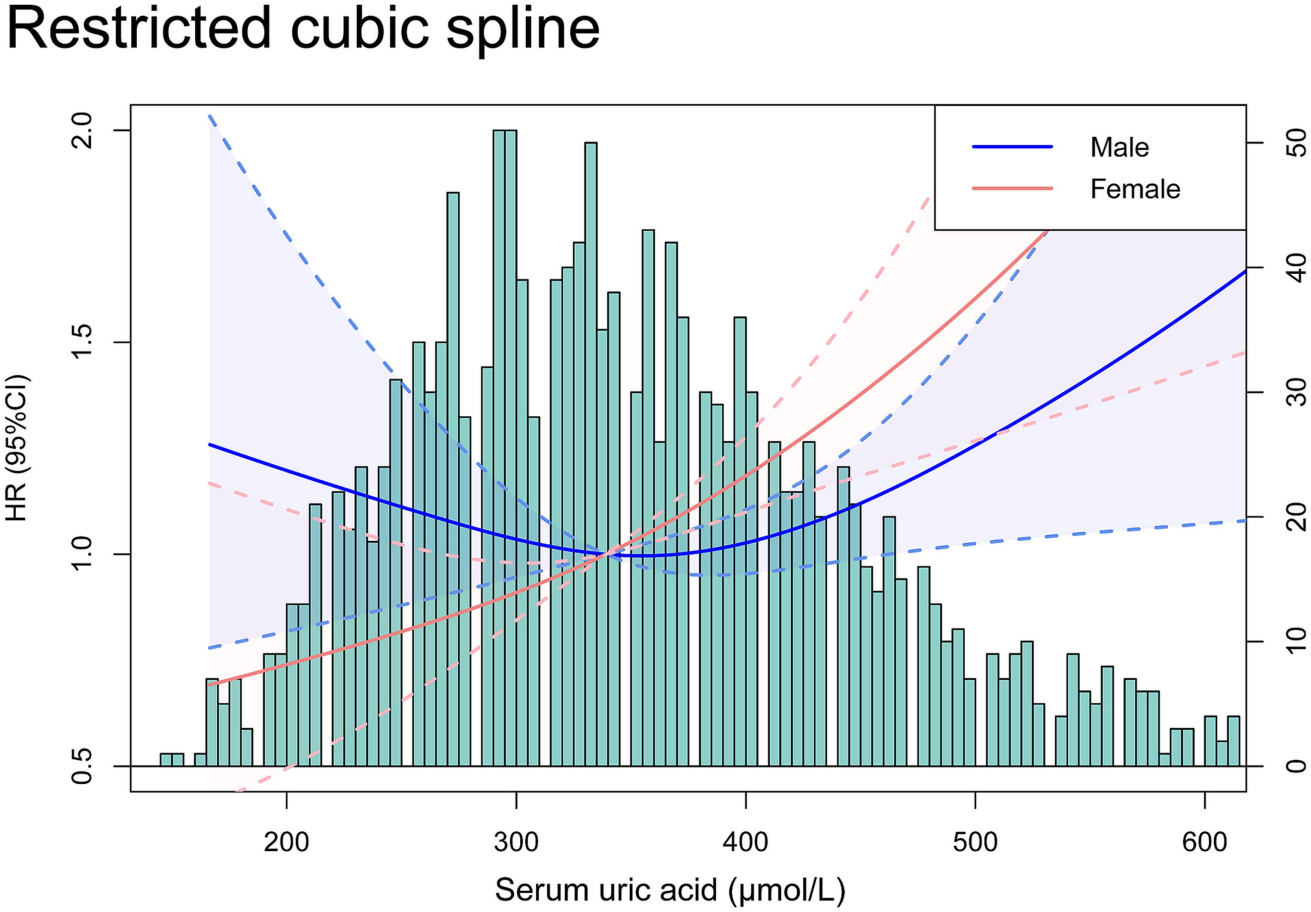
Restricted cubic spline curves analyzed the relationship between serum uric acid and the risk of all-cause mortality in stroke patients by gender.

Time-dependent ROC curve analyses (Figure 3) further reinforced these findings. The fully adjusted Model 3 had higher AUCs in predicting 3-year, 5-year, and 10-year mortality rates compared to the unadjusted model, demonstrating superior predictive performance. This indicates that hyperuricemia is an independent strong predictor of long-term mortality in stroke patients, and its predictive efficacy is more pronounced after comprehensive covariate adjustment. Additionally, RCS analyses (Figure 4) showed no significant nonlinear association between serum uric acid levels and stroke mortality risk in different genders (male *P* for nonlinearity = 0.0904; female *P* for nonlinearity = 0.6594), suggesting that stroke mortality risk may change in a relatively linear manner with uric acid levels.

### Subgroup analysis and mediation analysis

**Figure 5:**
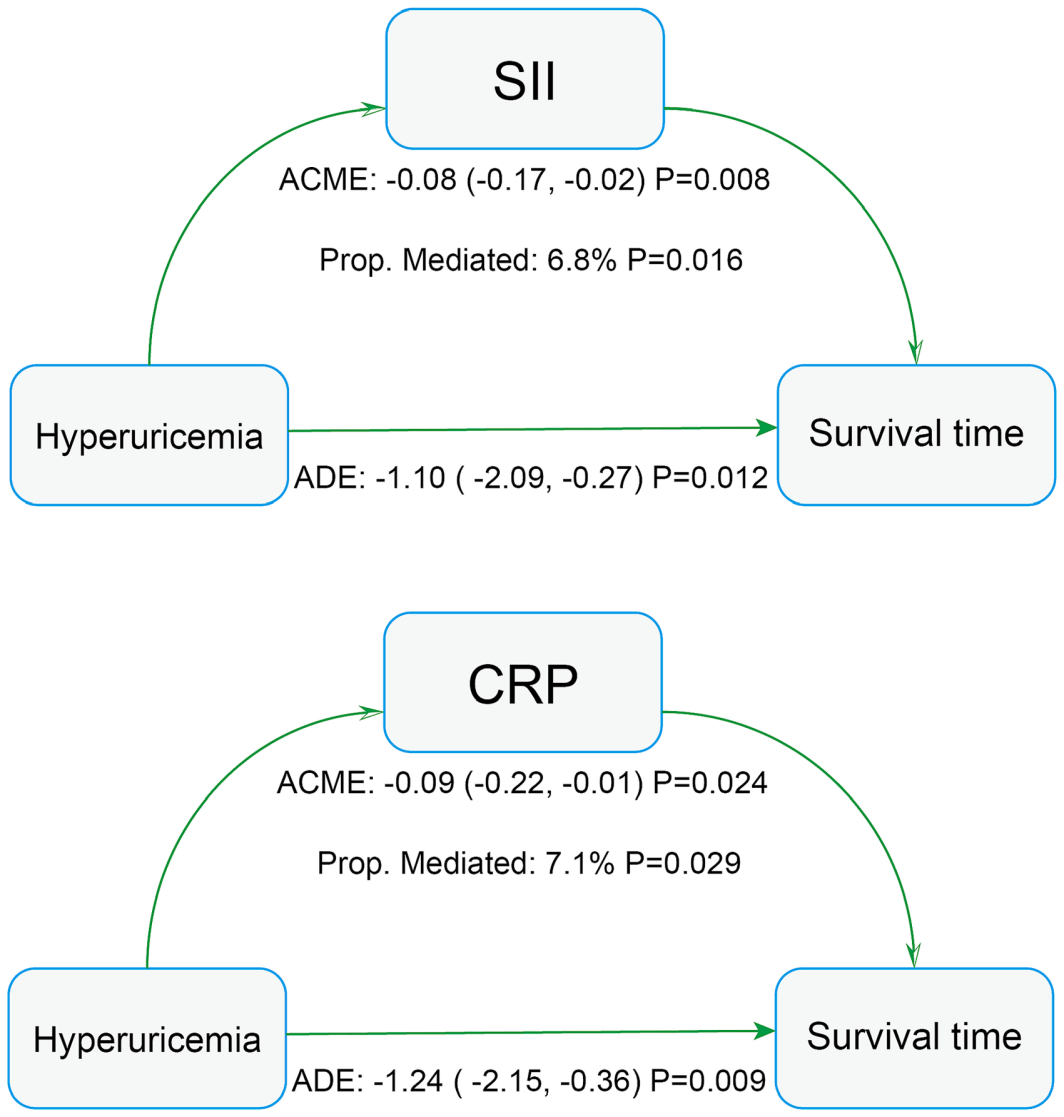
Mediation analysis of all-cause mortality in stroke patients. AST, aspartate aminotransferase; SII, systemic immune-inflammation index; CRP, C-reactive protein.

The increase in HR in the fully adjusted Cox model may reflect interactions between hyperuricemia and specific subgroups or indirect effects mediated through intermediary variables. Therefore, we conducted subgroup analyses and mediation analyses. Subgroup analysis revealed an absence of statistically significant interaction between hyperuricemia and all-cause mortality among stroke patients (*P* for interaction > 0.05 in all subgroups), indicating that the association between hyperuricemia and all-cause mortality was fairly stable across all subgroups (Table 5). The mediation analysis results (Figure 5) showed that hyperuricemia had a significant mediation effect through SII (ACME: -0.08, 95% CI: −0.17 to −0.02, *p* = 0.008) accounted for 6.8% of the total effect (*p* = 0.016), and CRP (ACME: -0.09, 95% CI: −0.22 to −0.01, *p* = 0.024) accounted for 7.1% of the total effect (*p* = 0.029). This suggests that hyperuricemia not only directly affects the mortality risk of stroke patients but also partially indirectly influences survival time through SII and CRP.

**Table 5:** Subgroup analysis of all-cause mortality in stroke patients.

Variable	HR (95%CI)	*p* value	*P* for interaction
Sex			0.302
Female	1.41 (1.09, 1.83)	0.01	
Male	1.12 (0.89, 1.41)	0.329	
Age			0.224
Age < 50	1.82 (0.47, 6.98)	0.384	
Age 50–65	1.29 (0.80, 2.10)	0.295	
Age > 65	1.27 (1.06, 1.53)	0.009	
Race			0.305
Mexican American	1.13 (0.48, 2.65)	0.772	
Non-Hispanic Black	1.50 (1.04, 2.17)	0.029	
Non-Hispanic White	1.26 (1.02, 1.55)	0.033	
Other	0.78 (0.34, 1.81)	0.564	
Poverty level			0.981
Non-poverty	1.26 (1.05, 1.52)	0.015	
Poverty	1.29 (0.85, 1.96)	0.228	
Marital status			0.650
No	1.30 (1.02, 1.64)	0.033	
Yes	1.15 (0.90, 1.47)	0.263	
Education level			0.473
Below high school	1.20 (0.92, 1.57)	0.180	
High School or above	1.29 (1.04, 1.61)	0.021	
Smoking			0.453
No	1.29 (1.07, 1.54)	0.006	
Yes	1.11 (0.69, 1.78)	0.681	
Drinking			0.748
No	1.29 (1.02, 1.63)	0.035	
Yes	1.23 (0.97, 1.57)	0.092	
Hypertension			0.271
No	1.67 (1.03, 2.72)	0.039	
Yes	1.23 (1.03, 1.47)	0.024	
CHD			0.718
No	1.25 (1.03, 1.51)	0.025	
Yes	1.20 (0.85, 1.71)	0.301	
Diabetes			0.589
No	1.28 (1.03, 1.60)	0.028	
Yes	1.20 (0.93, 1.56)	0.168	
Cancer			0.321
No	1.29 (1.06, 1.57)	0.013	
Yes	1.19 (0.86, 1.63)	0.294	

The regression model adjusted covariates as in Model 3. CHD, coronary heart disease; HR, hazard rate; CI, confidence interval.

### Sensitive analysis

In the sensitivity analyses, the unweighted logistic regression results (Supplementary Table S1) showed that serum uric acid and hyperuricemia were significantly associated with the occurrence of stroke. Including potential confounding factors such as asthma, liver disease, and hyperlipidemia in the final adjustment model (Supplementary Table S2), and excluding patients with survival times less than 12 months (Supplementary Table S3), did not significantly change the association between hyperuricemia and mortality in stroke patients. Furthermore, treating serum uric acid as a continuous variable was associated with all-cause mortality and cardiovascular disease mortality in stroke patients. However, in the quartile analysis, only the highest quartile (SUA > 410.4 μmol/L) showed a statistically significant association with all-cause mortality in stroke patients (Supplementary Table S4).

## Discussion

This study is the largest sample size investigation to date based on NHANES data examining the association between uric acid and stroke prevalence, as well as mortality risk in stroke patients. We found that higher uric acid levels are associated with stroke prevalence, consistent with previous studies from various cohorts (23–25). A single-center emergency study has confirmed that serum uric acid levels are significantly elevated in stroke patients (especially those with ischemic stroke) presenting to the emergency department. The study also found that hyperuricemia increased the risk of ischemic stroke by 2.4 times, while hypouricemia reduced the risk. This suggests that monitoring uric acid levels in the emergency setting holds potential value for identifying stroke, particularly ischemic stroke (26). Furthermore, hyperuricemia was associated with increased mortality risk in stroke patients. The Kaplan–Meier survival curves showed that stroke patients with hyperuricemia had significantly reduced survival rates. Cox regression analysis further confirmed this finding; even after adjusting for multiple covariates, hyperuricemia remained an independent predictor of mortality.

It remains controversial, however, whether SUA levels affect stroke prognosis. Some studies have shown a significant positive correlation between SUA levels and the prognosis of ischemic stroke (27, 28) with a greater impact observed in women (29). Higher SUA levels pose a higher mortality risk in elderly women over 50 years old (30) and some studies have identified it as an independent predictor of stroke in elderly postmenopausal women (31), which aligns with our results (steeper RCS in women and higher HR in female patients in subgroup analysis). Conversely, other studies have shown no significant correlation between serum uric acid levels and ischemic stroke prognosis (32) and some have even suggested that higher uric acid levels have neuroprotective effects after acute ischemic stroke (19, 20). Given the longer follow-up time in NHANES data, our results tend to reveal the association between hyperuricemia and long-term poor prognosis in stroke patients, enabling a more thorough understanding of SUA levels’ impact on stroke prognosis.

The potential mechanisms underlying the association between hyperuricemia and increased mortality in stroke patients are multifaceted. First, elevated serum uric acid levels may lead to endothelial dysfunction (33, 34) and oxidative stress (35). Importantly, our mediation analysis specifically identified systemic inflammation (measured by SII and CRP) as a significant pathway linking hyperuricemia to mortality. This finding aligns with substantial evidence linking hyperuricemia to pro-inflammatory states (36, 37), which can directly damage the vasculature and promote adverse outcomes. The significant mediation effects through SII and CRP provide empirical support for the role of inflammation in this association. Moreover, uric acid may exacerbate stroke prognosis by altering platelet reactivity (38) and promoting vascular calcification (39). The endothelial dysfunction pathway, suggested by uric acid’s link to reduced nitric oxide production, is also a plausible contributor to mortality risk, although it was not explicitly captured as a mediator in our current analysis. The metabolic syndrome (including hypertension, insulin resistance, and dyslipidemia) and hyperuricemia are also related (40), which may further worsen stroke outcomes through complex interactions involving the mechanisms described above.

Notably, the slight increase in HR in the fully adjusted model indicates a robust association between hyperuricemia and poorer survival outcomes. Mediation analysis further showed that SII and CRP partially mediated the effect of hyperuricemia on mortality in stroke patients. The inflammatory marker SII, composed of platelet count, neutrophil count, and lymphocyte count (41), is closely related to atherosclerosis and cardiovascular diseases (42, 43). CRP is a well-established inflammatory marker. Therefore, we suggest that inflammation plays a mediating role in this association. Additionally, when evaluating SUA quartiles, only the highest quartile (SUA > 410.4 μmol/L) was significantly associated with stroke mortality, which is close to the clinical threshold for defining hyperuricemia, indicating that patients with extremely high serum uric acid levels may face a higher risk of stroke mortality.

However, this study has several limitations. First, stroke diagnosis relied on self-reported questionnaire data. Although this approach is practical for large-scale cohort studies, it is susceptible to misclassification: participants may underreport stroke events due to unrecognized clinical symptoms (e.g., silent strokes), recall bias, or misinterpretation of questions, potentially leading to an underestimation of the true effect size. Second, SUA levels were based on a single measurement. SUA concentration exhibits within-individual variability influenced by factors such as recent diet (e.g., purine intake) and medication use (e.g., diuretics, allopurinol). A single measurement may fail to accurately capture an individual’s long-term average SUA exposure level during the critical period preceding the stroke event. As with all observational studies, not all potential confounding factors could be adjusted for in the statistical model; unmeasured confounders (such as urate-lowering medication information not directly available in the NHANES database) may still influence the results. The inflammatory biomarkers assessed in this study (CRP and SII), while biologically significant and clinically useful, do not encompass a broader range of markers specific to particular inflammatory pathways (such as specific cytokines, adhesion molecules, etc.). These additional markers could potentially provide deeper mechanistic insights. Future research should incorporate more comprehensive inflammatory profiling to further elucidate the specific inflammatory mechanisms through which uric acid influences stroke risk. Although our mediation analysis suggested a potential mediating role of inflammation in the association between SUA and stroke, it is imperative to emphasize that analyses based on observational data cannot definitively establish causality. More precise experimental or longitudinal studies are needed to confirm the mediating role of inflammation and further elucidate its underlying mechanisms. The findings of this study are based on NHANES data, with its national representativeness being its core strength. However, further validation is needed in other independent populations, particularly diverse national or ethnic populations, in the future. This study acknowledges the potential confounding effects of urate-lowering medications and other substances on serum uric acid levels. However, due to structural limitations in NHANES medication data — including non-continuous assessment of medication history (captured only within 30-day recall windows), absence of dosage/duration information, and significant rates of missing data and underreporting of over-the-counter medications — reliable adjustment for these confounders was not feasible. This limitation, common to observational studies utilizing NHANES, is explicitly noted to contextualize our findings. Future prospective studies with longitudinal medication records are warranted to validate these associations. Finally, due to the nature of NHANES data, we were unable to differentiate between ischemic stroke and hemorrhagic stroke. Future prospective studies with rigorous subtype determination (e.g., based on neuroimaging) and larger stroke cohorts are needed to investigate potential differential mechanisms.

## Conclusion

In this study, we found that hyperuricemia is significantly associated with increased prevalence of stroke among U.S. adults and is an independent predictor of higher all-cause and cardio-cerebrovascular mortality in stroke patients. Importantly, the mediation analysis indicates that inflammation partially mediates this association. Our study underscores the importance of monitoring and managing SUA levels to improve stroke outcomes. We recommend further research to explore the underlying biological mechanisms and to evaluate whether interventions targeting hyperuricemia could reduce mortality in stroke patients.

## Data Availability

Publicly available datasets were analyzed in this study. This data can be found at: https://www.cdc.gov/nchs/nhanes/.

## Glossary

NHANES: National Health and Nutrition Examination Survey
SUA: Serum Uric Acid
CVDs: Cardiovascular Diseases
BMI: Body Mass Index
TC: Total Cholesterol
AST: Aspartate Aminotransferase
ALT: Alanine Aminotransferase
CHD: Coronary Heart Disease
ROC: Receiver Operating Characteristic
AUC: Area Under the Curve
HR: Hazard Ratio
CI: Confidence Interval
ACME: Average Causal Mediation Effect
ADE: Average Direct Effect
SD: Standard Deviation
CRP: C-reactive Protein
SII: Systemic Inflammatory Index
RCS: Restricted Cubic Spline
KM: Kaplan–Meier
P: *p*-value
