# Supplementary material for: Inflammation mediates the association between hyperuricemia and stroke mortality: a cohort study
Source: Front Neurol. 2025 Aug 1;16:1599730. doi: 10.3389/fneur.2025.1599730 (PMC12354573; doi:10.3389/fneur.2025.1599730)
Supplement: Supplementary file 1 [file Data_Sheet_1.docx]

Inflammation mediates the association between hyperuricemia and stroke mortality: a cohort study

Table S1 Unweighted logistic regression.

| **Exposure** | **Model 1** | | **Model 2** | | **Model 3** | |
| --- | --- | --- | --- | --- | --- | --- |
|  | **OR (95%CI)** | **P value** | **OR (95%CI)** | **P value** | **OR (95%CI)** | **P value** |
| log (SUA) | 2.95 (2.43, 3.58) | <0.001 | 1.84 (1.50, 2.27) | <0.001 | 1.38 (1.11, 1.71) | 0.003 |
| Non-hyperuricemia | reference | | reference | | reference | |
| Hyperuricemia | 1.99 (1.79, 2.22) | <0.001 | 1.46 (1.30, 1.63) | <0.001 | 1.24 (1.10, 1.39) | <0.001 |

Model 1: Did not adjust any covariates.

Model 2: Adjusted for age, sex, and race.

Model 3: Adjusted for sex, age, race, poverty level, marital status, education level, smoking, drinking, hypertension, coronary heart disease, diabetes, cancer, body mass index, total cholesterol, aspartate aminotransferase, alanine aminotransferase, albumin, monocytes, neutrophils, lymphocytes, and platelets.

OR: odds ratio, CI: confidence interval, SUA: serum uric acid.

Table S2 Updated adjusted model.

| **Exposure** | **HR (95%CI)** | |
| --- | --- | --- |
|  | **All-cause mortality** | **CVDs mortality** |
| Non-hyperuricemia | reference | reference |
| Hyperuricemia | 1.24 (1.05, 1.47) | 1.38 (1.05, 1.81) |

Adjusted for sex, age, race, poverty level, marital status, education level, smoking, drinking, hypertension, coronary heart disease, diabetes, cancer, asthma, liver disease, hyperlipidemia, body mass index, total cholesterol, aspartate aminotransferase, alanine aminotransferase, albumin, monocytes, neutrophils, lymphocytes, and platelets. HR: hazard rate, CI: confidence interval, CVDs: cardio-cerebrovascular diseases.

Table S3 Exclusion of data with too short a survival time (1 year).

| **Exposure** | **HR (95%CI)** | |
| --- | --- | --- |
|  | **All-cause mortality** | **CVDs mortality** |
| Non-hyperuricemia | reference | reference |
| Hyperuricemia | 1.25 (1.04, 1.49) | 1.30 (1.01, 1.72) |

Adjusted for sex, age, race, poverty level, marital status, education level, smoking, drinking, hypertension, coronary heart disease, diabetes, cancer, body mass index, total cholesterol, aspartate aminotransferase, alanine aminotransferase, albumin, monocytes, neutrophils, lymphocytes, and platelets. HR: hazard rate, CI: confidence interval, CVDs: cardio-cerebrovascular diseases.

Table S4 Association of serum uric acid levels with mortality risk in stroke patients.

| **Exposure** | **HR (95%CI)** | |
| --- | --- | --- |
|  | **All-cause mortality** | **CVDs mortality** |
| log (SUA) | 1.77 (1.29, 2.43) | 1.90 (1.13, 3.18) |
| SUA Quartile 1 | reference | reference |
| SUA Quartile 2 | 1.02 (0.80, 1.29) | 0.78 (0.52, 1.17) |
| SUA Quartile 3 | 1.07 (0.84, 1.36) | 0.94 (0.64, 1.39) |
| SUA Quartile 4 | 1.44 (1.14, 1.83) | 1.36 (0.93, 2.00) |
| P for trend | 0.002 | 0.04 |

Adjusted for sex, age, race, poverty level, marital status, education level, smoking, drinking, hypertension, coronary heart disease, diabetes, cancer, body mass index, total cholesterol, aspartate aminotransferase, alanine aminotransferase, albumin, monocytes, neutrophils, lymphocytes, and platelets. SUA Quartiles Q1: < 279.6; Q2: 279.6- 339.0; Q3: 339.0-410.4; Q4: >410.4. HR: hazard rate, CI: confidence interval, CVDs: cardio-cerebrovascular diseases, SUA: serum uric acid.
